# Multi-Input Dual-Stream Capsule Network for Improved Lung and Colon Cancer Classification

**DOI:** 10.3390/diagnostics11081485

**Published:** 2021-08-16

**Authors:** Mumtaz Ali, Riaz Ali

**Affiliations:** 1 School of Computer Science, Huazhong University of Science and Technology, Wuhan 430074, China; 2 Department of Computer Systems Engineering, Sukkur IBA University, Sukkur 65200, Pakistan; 3 Department of Computer Science, Sukkur IBA University, Sukkur 65200, Pakistan; riaz.khp@iba-suk.edu.pk

**Keywords:** lung cancer, colon cancer, histopathological images, multi-input capsule networks

## Abstract

Lung and colon cancers are two of the most common causes of death and morbidity in humans. One of the most important aspects of appropriate treatment is the histopathological diagnosis of such cancers. As a result, the main goal of this study is to use a multi-input capsule network and digital histopathology images to build an enhanced computerized diagnosis system for detecting squamous cell carcinomas and adenocarcinomas of the lungs, as well as adenocarcinomas of the colon. Two convolutional layer blocks are used in the proposed multi-input capsule network. The CLB (Convolutional Layers Block) employs traditional convolutional layers, whereas the SCLB (Separable Convolutional Layers Block) employs separable convolutional layers. The CLB block takes unprocessed histopathology images as input, whereas the SCLB block takes uniquely pre-processed histopathological images. The pre-processing method uses color balancing, gamma correction, image sharpening, and multi-scale fusion as the major processes because histopathology slide images are typically red blue. All three channels (Red, Green, and Blue) are adequately compensated during the color balancing phase. The dual-input technique aids the model’s ability to learn features more effectively. On the benchmark LC25000 dataset, the empirical analysis indicates a significant improvement in classification results. The proposed model provides cutting-edge performance in all classes, with 99.58% overall accuracy for lung and colon abnormalities based on histopathological images.

## 1. Introduction

The World Health Organization considers cancer being one of the deadliest diseases. Lung cancer is responsible for 18.4% of cancer-related deaths and 11.6% of all cancer cases. In the same way, colon cancer accounts for 9.2% of all cancer-related fatalities worldwide [1,2,3]. Globally, there has been an increase in recent trends for malignant tumor rates, which could be attributed to an increase in population. Cancer affects people of all ages, but those between the ages of 50 and 60 are the most vulnerable. According to some estimates, death rates could rise by 60% by 2035 if current trends continue [4,5].

Malignant cells arise when cells in the lungs begin to mutate uncontrollably, forming clusters known as tumors [6]. The rise in cancer incidence globally is attributable to several causes, the most important of which is increased lung exposure to hazardous substances and an increase in the population of elderly individuals. The symptoms of such ailments are typically undetectable until they have spread to other organs of the body, making treatment challenging [7].

Persons who smoke have a higher risk of acquiring lung cancer at some point in their lives, but lung cancer can also affect people who have never smoked. Adenocarcinoma and squamous cell carcinoma are the most prevalent kinds of lung cancer, while other histopathological categories include small and large cell carcinomas [8]. Adenocarcinoma is a type of lung cancer that can affect persons who smoke or have recently quit smoking, as well as those who do not. It primarily affects women and young people, and it usually starts in the outer layers of the lungs before swiftly spreading. Squamous cell carcinomas can develop in any area of the lungs and are found in people who smoke or have smoked in the past. It spreads and expands at such a rapid rate that it is difficult to treat [9,10].

When healthy cells, as well as the lining of the rectum or colon, expand uncontrollably, a tumor forms. This type of tumor is usually malignant [11]. Adenocarcinomas of the rectum or colon commonly develop along the lining of the large intestine, starting in the epithelial cells and spreading to the other layers. Signet ring cell adenocarcinoma and mucinous adenocarcinomas are two less prevalent adenocarcinoma subtypes. The two subtypes, however, are difficult to treat since they are highly aggressive [12]. Gender, ethnicity, age, smoking habits, and financial situation can all have an impact on how your body ages. However, if a person has a rare genetic condition, mutations can occur in as little as a few months [13].

Automated systems based on deep learning to diagnose health conditions specially cancers have become a norm in recent times. There are various works that attempt to automate such diagnosis, although the majority of them rely on CT and MRI images [14,15,16]. The early prediction of breast cancer for instance based on dynamic contrast-enhanced magnetic resonance imaging (DCE-MRI) has significantly been improved using deep learning approaches [17]. Similarly, CT scans have been used for brain tumor image classification and lung cancer detection with deep learning [18].

Deep learning based diagnosis of lung and colon cancers have been increasingly prominent research subjects in recent years. Most successful studies have used histopathology slide images to aid in automated diagnosis. To diagnose lung and colon cancers automatically, this study relies solely on histopathological images. This work focuses to classify the images for lung and colon cancer into five classes: (1) Squamous cell carcinomas (2) Adenocarcinomas (3) Benign lung tumors (4) Adenocarcinomas (5) Benign colon tumors. The main objective of the current work is to effectively improve the deep learning based diagnosis of such lung and colon cancers by providing better results.

Convolutional neural networks have been used in practically almost all deep learning algorithms for medical image classification since their re-emergence [19,20,21]. There are two fundamental forms of convolutional neural networks, each with a somewhat different working principle. Conventional convolutional neural networks and separable convolutional neural networks are the two varieties. Capsule Networks, on the other hand, are gaining popularity in medical image classification due to their lightweight models [22,23]. As a result, in this research, we use a hybrid strategy to classify lung and colon cancer histopathology slide images by combining conventional convolutional layers [24], separable convolutional layers [25], and a capsule network [26]. Pre-processing the images to balance the colors and maintain overall detail for better feature learning is a crucial aspect of this research, in addition to the proposed network.

To complete the task, we present a multi-input deep learning model based on capsule networks. There are two input streams in the proposed approach. The first is based on images from unprocessed histopathology slides, while the second is based on images from pre-processed histopathological slides. The input is received by two independent blocks of convolutional layers, CLB and SCLB. Unprocessed images are accepted by the CLB, whereas pre-processed images are accepted by the SCLB. CLB and SCLB blocks are linked to primary capsule layers to complete the classification process. Pre-processing is employed to balance RGB channels since histopathological slide images have a red bluish appearance. This kind of pre-processing helps with feature learning, which enhances overall classification performance.

Following are the major contributions of this paper:

- Propose a novel multi-input capsule network to classify lung and colon tumors into five categories: squamous cell carcinomas, adenocarcinomas, and benign for the lung, and adenocarcinomas and benign for the colon.
- Enhance feature learning of the deep learning models by pre-processing histopathological slide images by sharpening, gamma correction and multi-scale fusion.
- Present state-of-the-art results for the classification of histopathological slide images for automated diagnosis of lung and colon cancer.

## 2. Related Work

The researchers have always been influenced by the type of medical imaging data while developing deep learning-based diagnosis and prognosis systems. CT scans, X-rays, MRIs, Endoscopic and histopathology slides are the most prevalent medical imaging data [27,28,29]. Because cancer is one of the deadliest and most complex diseases, researchers have found it difficult to automate its classification and detection. The type of cancer and the organ where cancer has originated makes its detection more difficult. Despite the complexity of the problem, there are significant contributions where the authors have used deep learning methods to automate cancer detection systems [30].

Most prevalent types of cancers such as breast cancer can be detected with deep learning systems. For instance, the authors Houssami et al. [31] and Rakhlin et al. [32] devise deep learning methods to detect breast cancer with reasonable accuracy. Similarly, the authors, Lorencin et al. [33,34] use deep learning methods to diagnose cancer in the urinary bladder. Skin cancer is another leading type of cancer and there are few major contributions, for instance, Jinnai et al. [35] present a deep learning method to detect skin cancer. Deep learning methods have also been used for detection of cancer stem cell morphology [36], gastric cancer [37] and grading of oral squamous cell carcinoma [38].

Deep learning algorithms for classifying and diagnosing lung and colon cancer using histopathology images have become a popular research topic in recent years [39], however, due to a paucity of data, no substantial progress has been achieved so far [40]. Despite the lack of data, a few authors have contributed significantly [41]. The studies that use data produced from histopathology slides are the focus of this section since we are solely interested in data derived from histopathology slides in this study.

Some authors focused entirely on lung cancer classification [42], while others primarily focused on colon cancer classification [43]. Researchers in recent works have attempted to classify images of lung and colon cancer at the same time. In terms of methodology, the authors have either employed pre-trained models in a transfer learning setting or trained their own designed models from scratch [44,45].

For only the lung cancer classification there are few notable works, for instance, Abbas et al. [46] use pre-trained models VGG-19, AlexNet, ResNet-18, ResNet-34, ResNet-50 and ResNet-101 for classification of only the lung cancers. They classify images into three categories of squamous cell carcinoma-lung, adenocarcinoma-lung and benign-lung. They claim to achieve f1-scores of of 97.3%, 99.7%, 98.6%, 99.2%, 99.9% and 99.9% for all the pre-trained models AlexNet, VGG-19, ResNet-18, ResNet-34, ResNet-50 and ResNet-101 respectively. Roy et al. [47], on the other hand, classify lung cancer histopathology images using a capsule network. They claim to attain 99% accuracy using a fairly standard setup.

There have been a few important contributions to the classification of colon cancer. To classify histopathological images of colonic tissue, Bukhari et al. [48] use three architectures of convolutional neural networks: ResNet-18, ResNet-30, and ResNet50. They claim that the models ResNet-30 and ResNet-18 each achieve 93.04% accuracy, while ResNet-50 achieves 93.91% accuracy.

Masud et al. [43] classify histopathological lung and colon images using a novel deep learning-based technique. They used domain transformations of two types to extract four sets of features for image classification. They then combine the features of both categories to arrive at final classification findings. They claim to achieve an accuracy of 96.33%. Similarly, Sanidhya et al. [2] classify histopathological images into five categories: squamous cell carcinomas, adenocarcinomas, benign lung images, adenocarcinomas, and benign colon images, using a shallow neural network architecture. They claim to have achieved 97% and 96% accuracy in lung and colon cancer classifications, respectively, in their research.

In nutshell, all of the modern-day deep learning techniques primarily focus on histopathological images and they need a drastic improvement to produce the best results. Most of the prevailing techniques employ methods for abnormalities detection on either lung or colon tissues. Whereas there’s need for a version that could produce better results for the abnormalities at each of the organs.

## 3. Method

**Figure 1:**
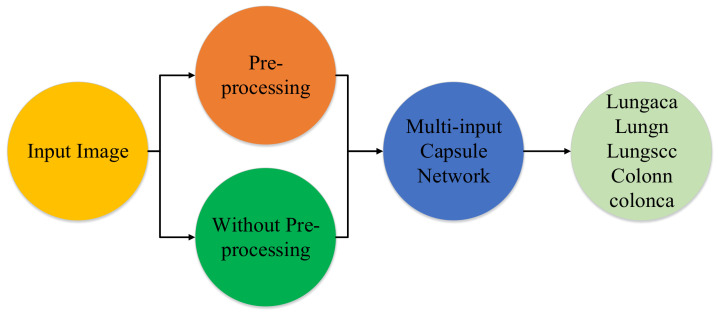
Diagram depicting the method in a very brief manner. lungaca: lung adenocarcinomas, lungscc: lung squamous cell carcinomas, lungn: lung benign, colonca: colon adenocarcinomas, and colonn: colon benign.

The proposed model’s main goal is to identify lung and colon cancer in *N* training histopathology images by classifying them into five classes: Squamous cell carcinomas, Adenocarcinomas, Benign lung tumors, Adenocarcinomas, and Benign colon tumors. To accomplish this, a unique multi-input dual-stream capsule network is used. It is necessary to briefly discuss the important components of the proposed model’s fundamental architecture. Overall there are three major parts of the proposed method which are the proposed model, the threefold margin loss and the unique pre-processing method. Figure 1 shows the overall process of the proposed approach. In this section, we present the proposed model, the threefold margin loss and the pre-processing method. Section 3.1 presents the proposed model and its key components, Section 3.2 presents the threefold margin loss and Section 3.3 describes the procedure of the proposed pre-processing method.

### 3.1. Proposed Network and Its Key Components

The proposed network is based on a multi-input dual-stream network, and it uses conventional convolutional layers, separable convolutional layers and primary capsule layers. Fundamentally, convolutional layers belong to convolutional neural networks (CNNs) [49], separable convolutional layers belong to depthwise separable convolutional neural networks [25] and primary capsule layers belong to capsule networks [50], therefore, we briefly describe these fundamental concepts before describing the overall architecture of the proposed model.

#### 3.1.1. Convolutional Neural Networks

One of the most prominent approaches in deep learning-based medical image classification algorithms is convolutional neural networks. The fundamental purpose of CNNs is to learn features for patterns inside images, which aids recognition and classification. In general, any image, such as images of human faces, landmarks, trees, or plants, or simply aspects of any kind of visual data, can be employed as an input image for CNNs [51]. CNNs learn features based on appropriate parameters and their related optimal values from a series of training images. In a CNN network, there are three different sorts of layers:

(1) Convolutional layers: These layers are made up of a number of nodes that extract important information from the input images. This sort of layers employ a large number of kernels/filters to achieve the main goal of feature learning on input images.
(2) Pooling layers: After convolutional layers, these layers are frequently employed. The main purpose of these layers is to minimize the spatial dimension (width and height) of the input data before passing it on to the following layers. These layers aid in the computational efficiency of CNN models.
(3) Fully-connected layers: This types of layers are fully connected to the output of the CNN network’s preceding layers. These layers aid in the learning of output probabilities, which are then used to determine the model’s accuracy. The mathematical formulation of convolution (C) is given as follows:
  (1) $$C{\left(W,y\right)}_{\left(i,j\right)}=\sum_{k,l,m}^{K,L,M}W_{\left(k,l,m\right)}\cdot y_{\left(i+k,j+l,m\right)}$$
  here the *K* and *L* represent the width and the height of the input whereas the *M* represents the number of filters. Similarly, *W* and *y* denote the input and the output respectively.

#### 3.1.2. Depthwise Separable Convolutional Neural Netwoks

Generally, there are two types of separable convolutions in separable convolutional neural networks, named spatial separable convolutions (SC), and depthwise separable convolutions. We in this paper are using depthwise separable convolutions, and operation of depthwise convolutions may be considered as grouped convolutions or in the form of “inception modules” which were used in the architecture of Xception [25]. It is based on a spatial convolution which is executed independently on every input channel. A pointwise convolution is performed after the spatial convolution that is a conventional convolution operation by using 1×1 windows, subsequently, a newer channel space emerges due to the projection of the channels computed during depthwise convolution.

The mathematical formulation of depthwise convolution (DC) and pointwise convolution (PC) is given as follows:

(2) $$PC{\left(W,y\right)}_{\left(i,j\right)}=\sum_{m}^{M}W_{m}\cdot y_{\left(i,j,m\right)}$$

(3) $$DC{\left(W,y\right)}_{\left(i,j\right)}=\sum_{k,l}^{K,L}W_{\left(k,l\right)}⊙y_{\left(i+k,j+l\right)}$$

(4) $$SC{\left(W_{p},W_{d},y\right)}_{\left(i,j\right)}=PC_{\left(i,j\right)}\left(W_{p},DC_{\left(i,j\right)}\left(W_{d},y\right)\right)$$

here $W_{p}$ and $W_{d}$ are the input for pointwise and depthwise convolution respectively. The symbol ⊙ in the Equation (3) refers to element-wise product. As a result, the core concept behind depthwise separable convolutions would be to divide the feature learning accomplished by standard convolutions over a combined “space-cross-channels domain” into 2 phases: spatial feature learning and channel combination. If the 2D or 3D inputs that convolutions operate on have both fairly autonomous channels and strongly linked spatial locations, as is usually assumed, this is a significant generalisation.

#### 3.1.3. Capsule Networks

In Capsule Networks [50], the capsules are a set of neurons where activity vectors include numerous orientation properties as well as their length. The probability of a certain entity’s existence is represented by these activity vectors. Because the pooling layers are the weak links in CNNs, these processes could easily remove or dilute image features, causing basic object structures to be disrupted [52]. Therefore, they are substituted by a more suitable procedure known as “routing-by-agreement”. The outputs are received by parent capsules in the following layers based on this rule, however, their coupling coefficients differ. Each capsule tries to produce an output that is as similar to the parent capsule’s output as possible; if they succeed, the coupling coefficient between these capsules increases [53]. Let $u_{i}$ be the output of the *i*th capsule, its predicted output for the *j*th parent capsule can be acquired as:

(5) $${\hat{u}}_{u|i}=W_{ij}u_{i},$$

here the ${\hat{u}}_{u|i}$ serves as the vector which is output of the capsule number *j* computed by capsule *i* in a lower layer. Similarly, $W_{ij}$ is learned through a backward pass and it is called as weight matrix. On the basis of compatibility between parent capsule and the capsules in lower layer the coupling coefficients $c_{ij}$ are computed as:

(6) $$c_{ij}=\frac{exp(b_{ij})}{\sum_{k}exp\left(b_{ik}\right)},$$

here the term $b_{ij}$ is the logarithmic probability which indicates whether the capsule *j* should be coupled with capsule *i* or not. Initially the value of $b_{ij}$ is set to zero during the process of routing by agreement. Hence, the iput vector for the capsule *j* (which is a parent capsule) is computed as below:

(7) $$s_{j}=\sum_{i}c_{ij}{\hat{u}}_{j|i}.$$

Basically, to limit Capsule outputs from surpassing one and to construct the final result of each Capsule depending on its initial vector value defined in Equation (7) the following non-linear squashing function is applied.

(8) $$v_{j}=\frac{\left|\right|s_{j}{\left|\right|}^{2}}{1+\left|\right|s_{j}{\left|\right|}^{2}}\frac{s_{j}}{\left|\right|s_{j}\left|\right|},$$

where $v_{j}$ is the resultant output vector and $s_{j}$ is the input vector for the capsule *j*. Updating of log probabilities is only possible when $v_{j}$ and ${\hat{u}}_{j|i}$ are in agreement, and their inner product will be lager in such a case. Therefore, calculation of that agreement $a_{ij}$ is accomplished as follows:

(9) $$a_{ij}=v_{j}\cdot {\hat{u}}_{j|i}$$

#### 3.1.4. Proposed Multi-Input Dual-Stream Capsule Network

Initially, the capsule networks were solely designed to classify the images given in the MNIST [54] dataset, which contained images of the 28×28 dimension. The architecture of the network was based on a very basic network having 2 convolutional layers and a fully connected layer. The first layer had a stride of 1, there were only 256 channels along with the convolutional kernels of 9×9. The second layer was based on capsule layers called primary caps layers, it was having 32 channels and 8 convolutional capsules, which meant that each primary capsule containing eight convolutional units with a 9×9 kernel and a stride of 2 pixels. For both the layers the activation function was rectified linear unit (ReLU) [55]. The last layer had 16-dimensional capsules for each digit class, each of these capsules was designed to receive the inputs from all of the capsules.

Recently, a number of modifications of the originally proposed architecture have been developed, and these versions have been employed to address the underlying problem’s requirements. The processing cost and amount of trainable parameters have a significant impact on the output of a deep learning model. As a result, we tested a variety of architectures in this research, but we found that using a multi-input architecture, which combines the capabilities of traditional as well as separable convolutional layers with capsule layers, yielded the best results.

As capsule networks are traditionally made up of shared convolutional layers, primary capsule layers, and fully connected capsule layers in a standard approach. Conventional convolutions are used in the convolutional layers, but Separable convolutions have recently been demonstrated to be faster than regular convolutions. We wanted to employ two inputs for the model to get the benefits of separable and traditional convolutions, as well as a powerful pre-processing strategy that would be ideal for lung and colon cancer classification. Therefore, model is designed in that way to make it more robust.

**Figure 2:**
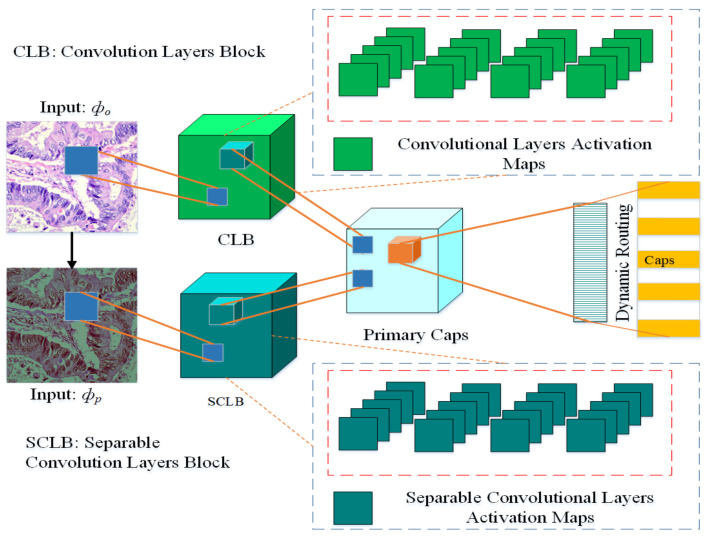
Proposed multi-input dual-stream capsule network, $\Phi_{o}$: Input images without pre-processing, $\Phi_{p}$: Input with pre-processed images. CLB: Convolutional Layers Block, SCLB: Separable Convolutional Layers Block.

The architecture of the proposed multi-input dual-stream capsule network is depicted in Figure 2, and summary of the proposed model is given in Table 1. The proposed model uses two inputs $\Phi_{o}$ and $\Phi_{p}$. The input $\Phi_{o}$ is directly from original data without any pre-processing other than size adjustment, the input $\Phi_{p}$ is the pre-processed version of the data. As it can be observed in the model, the blocks named CLB and SCLB receive the inputs $\Phi_{o}$ and $\Phi_{p}$ respectively. As a result, the model learns a variety of useful features that help it improve its performance.

More precisely, from inputs $\Phi_{o}$ and $\Phi_{p}$ the blocks CLB and SCLB learn the features denoted as $F_{CLB}$ and $F_{SCLB}$ respectively. These features are then combined as:

(10) $$F_{total}=\left(\begin{array}{c} F_{CLB}\\ F_{SCLB} \end{array}\right)$$

The features $F_{CLB}$ and $F_{SCLB}$ are then passed over to the primary capsule layers, the features here are reshaped and converted into vectors. These vectors are connected with fully connected capsules to learn the probabilities for the classification. Between the primary capsules layer and fully connected capsules layer, there is a mechanism called dynamic routing which serves as the bridge between these two layers.

The block CLB is based on four convolution layers, the channel is set at (64×64) with kernel size set at (3×3). Similarly, the block SCLB also has four layers but it is based on separable convolutional layers, there are also (64×64) channels and default kernel size is set at (3×3). At every successive layer, 64 feature maps are learned for convolutional and separable convolutional layers. The model learns 32 and 16 primary capsules for each stream respectively, after the merger, in total there are 48 primary capsules in the model.

The proposed model has a hybrid architecture, which logically has all the ingredients. For instance, it contains the capabilities of both types of convolutional layers (conventional and separable) and capsule layers. Similarly, it receives multiple inputs to learn rich features from an untouched original image and a pre-processed image.

### 3.2. Threefold Margin Loss

We use a novel threefold margin loss in this paper which has been explored after preliminary experiments. Formally, every capsule *k* inside the final layer has a loss function $l_{k}$ that assigns a high loss value to capsules with larger output initialization parameters whenever the entity doesn’t really exist. In our method we use multiple folds of the loss function in order to make it more robust. As there are two type of inputs for the model, therefore, to achieve optimal results the values of the given parameters in the loss function may play very important role. Hence, the threefold loss can be given has follows:

(11) $$\begin{array}{c} l_{k_{1}}=T_{k_{1}}max(0,m_{1}^{+}-\left|\right|v_{k_{1}}{\left||\right)}^{2}+\lambda_{1}\left(1-T_{k_{1}}\right)max(0,||v_{k_{1}}\left|\right|-m_{1}^{-}{)}^{2}\\ l_{k_{2}}=T_{k_{2}}max(0,m_{2}^{+}-\left|\right|v_{k_{2}}{\left||\right)}^{2}+\lambda_{2}\left(1-T_{k_{2}}\right)max(0,||v_{k_{2}}\left|\right|-m_{2}^{-}{)}^{2}\\ l_{k_{3}}=T_{k_{3}}max(0,m_{3}^{+}-\left|\right|v_{k_{3}}{\left||\right)}^{2}+\lambda_{3}\left(1-T_{k_{3}}\right)max(0,||v_{k_{3}}\left|\right|-m_{3}^{-}{)}^{2}\\ L_{T}=l_{k_{1}}+l_{k_{2}}+l_{k_{3}} \end{array}$$

### 3.3. Proposed Pre-Processing Method

The pre-processing of histopathological images consists of several steps. For instance, the objective of color balancing is to improve the aspect of images, by predominantly eliminating the unwanted color casting due to medium attenuation properties or different illumination. The perception of color in histopathological images is greatly related to depth, red-bluish appearance is the other problem that needs to be corrected. Almost all the methods of color balancing techniques estimate the color of the light source, subsequently, divide each color channel with the corresponding stabilized light source to acquire the required color consistency.

**Figure 3:**
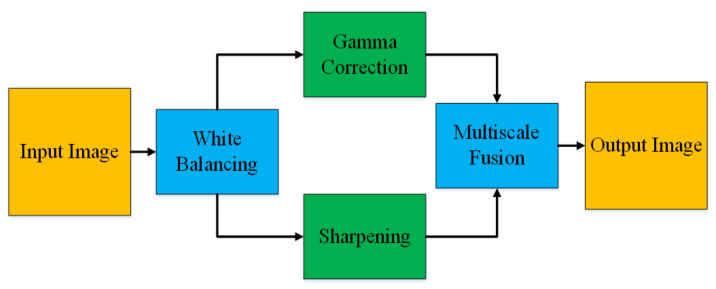
Image Enhancement.

**Figure 4:**
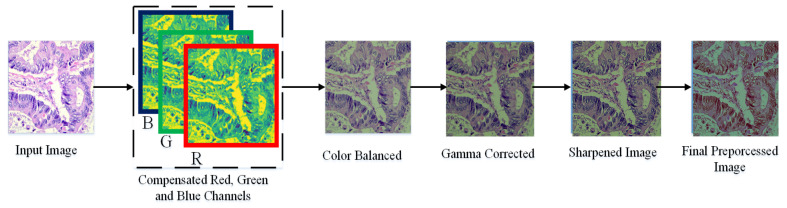
Image Enhancement procedure.

In this paper, we adopt a color balancing technique for digital images which was introduced in [56], where the authors try to compensate the red and green channels of the underwater images. Instead of compensating red and green channels only, we rather compensate all three channels to achieve the optimal results. Figure 3 and Figure 4 ilustrate the proposed pre-processing method. Mathematically at every location *x*, the red channel may be presented as:

(12) $$\Phi_{rc}\left(x\right)=\Phi_{r}\left(x\right)+\alpha \cdot \left({\bar{\Phi }}_{g}-{\bar{\Phi }}_{r}\right)\cdot \left(1-\Phi_{r}\left(x\right)\right)\cdot \Phi_{g}\left(x\right)$$

$\Phi_{r}$, $\Phi_{g}$ here symbolize the color channels of red and green of the image denoted as Φ. Both the channels fall within the interval [0,1] when they are normalized with the upper limit of the corresponding dynamic range. Similarly, ${\bar{\Phi }}_{r}$ and ${\bar{\Phi }}_{g}$ present the averaged value of $\Phi_{r}$ and $\Phi_{g}$. In the Equation (12), α symbolizes a constant parameter, whereas the factors in the second term originate from the previous observation. The authors in [56] reveal by testing that when α=1, we can get optimal results.

The blue channel $\Phi_{bc}$ can be compensated as follows:

(13) $$\Phi_{bc}\left(x\right)=\Phi_{b}\left(x\right)+\alpha \cdot \left({\bar{\Phi }}_{g}-{\bar{\Phi }}_{b}\right)\cdot \left(1-\Phi_{b}\left(x\right)\right)\cdot \Phi_{g}\left(x\right)$$

here $\Phi_{b}$, $\Phi_{g}$ epitomize the blue and green color channels of the image Φ, and α is set to 1. Similarly, the green channel is compensated as follows:

(14) $$\Phi_{gc}\left(x\right)=\Phi_{g}\left(x\right)+\alpha \cdot \left({\bar{\Phi }}_{r}-{\bar{\Phi }}_{g}\right)\cdot \left(1-\Phi_{g}\left(x\right)\right)\cdot \Phi_{r}\left(x\right)$$

where $\Phi_{g}$, $\Phi_{r}$ denote green and red color channels of the image Φ, and α is set to one. The step of the multi-scale fusion has two inputs, the input1 presents a gamma-corrected form of the images which are already color balanced. The unsharp masking technique is utilized for the image sharpening. To sharpen the original image, it is blended with the unsharpened image (which is Gaussian filtered). The unsharp masking formula which defined the unsharpened image is given by *S* as S=Φ+β(Φ−G*Φ). In this relation, Φ represents the candidate image to be sharpened, in our case it is the color-balanced image. The term G*Φ represents the Gaussian filtered form of Φ, and β symbolizes a parameter. Practically, the parameter β does not help to sharpen the Φ, nevertheless, when β has a too large value it results in regions that are over-saturated having highlights with very bright appearance and very dark shadows. To tackle this problem, we may present the sharpened images as below:

(15) S=(Φ+N{Φ−G*Φ})/2

N{.} here means the operator of linear normalization, which is sometimes referred to in the literature as the histogram stretching. During the process of fusion, different weights are used. Such weights are called Saliency Weight ($W_{S}$), Saturation Weight ($W_{Sat}$), and Laplacian Contrast weight ($W_{L}$). When the weight maps are used smartly during the process of blending it results in a better representation of the pixels with higher weights in the final image. The global contrast is estimated by ($W_{L}$) in the result of calculating the absolute value of the Laplacian filter which is applied to every luminance channel that is input. The ($W_{S}$) focuses on the salient objects which lose prominence. In this paper, the saliency estimator of Achantay et al. [57] is used to compute the level of saliency. The ($W_{Sat}$) helps the algorithm of the fusion to get used to the chromatic evidence by upholding extremely saturated regions. For each of the input $\Phi_{k}$, this weight map is solely calculated as a deviation for any pixel present at any location between the $R_{k}$,$G_{k}$ and $B_{k}$ color channels and the luminance $L_{k}$ of the *K*th input:

(16) $$W_{Sat}=\sqrt{1/3[{\left(R_{k}-L_{k}\right)}^{2}+{\left(G_{k}-L_{k}\right)}^{2}+{\left(B_{k}-L_{k}\right)}^{2}]}$$

Practically, three weight maps are combined into a single weight map for each input as follows. We can obtain an aggregated weight map $W_{k}$ by adding the weight maps $W_{L}$, $W_{S}$, and $W_{Sat}$ for each input. Then based on a pixel-per-pixel basis the aggregated maps are normalized by dividing each pixel’s weight map with the summation of that pixel’s weights on all the maps. The formal computation of the weight maps $W_{k}$ (which are already normalized) for each input as $\bar{W_{k}}=\left(W_{k}+\delta \right)/(\sum_{k=1}^{K}W_{k}+K\cdot \delta$). The δ symbolizes a term used for regularization which helps to make sure that all the inputs fairly contribute to the output. Throughout the study, the value of δ is set to 0.1. Based on the normalized weight maps, R(x) which is the reconstructed image may be obtained based on the fusion of the inputs defined with measures of the weight at each pixel location (x):

(17) $$R\left(x\right)=\sum_{k=1}^{K}{\bar{W}}_{k}\left(x\right)\Phi_{k}\left(x\right)$$

here the symbol $\Phi_{k}$ represents the input that is weighted with the normalized weight maps ${\bar{W}}_{k}$. The laplacian pyramid [58] is used for the multi-scale decomposition. A bandpass image is acquired with the help of the pyramid representation. In reality, the input image is filtered at each pyramid level by using a low-pass Gaussian kernel *G*, and the factor of 2 reduces the filtered image in both directions. An up-sampled version of the low-pass images is subtracted from the input image. Hence, the inverse of the Laplacian is approximated and a reduced lowpass image is used as the input for later levels of the pyramid.

Consequently, the decomposition of the inputs $\Phi_{k}$ results in a laplacian pyramid [58], whereas the Gaussian pyramid is used to decompose the normalized weight maps ${\bar{W}}_{k}$. There is the same number of levels in both the pyramids and the fusion of the inputs from both the pyramids is performed individualistically at the *L*:

(18) $$R_{l}\left(x\right)=\sum_{k=1}^{K}G_{l}\left\{W_{k}\left(x\right)\right\}L_{l}\Phi_{k}\left(x\right)$$

the symbol *l* here represents the levels of the pyramid and the symbol *k* denotes the count of the levels N which is dependent on the size of the image. It directly impacts the final quality of the visual perception of the combined image. The summation of all the levels of the fused contributions results in a dehazed image when it is appropriately up-sampled.

## 4. Experiments

### 4.1. Training the Proposed Model

#### 4.1.1. Training Dataset and Training Setup

We use the dataset named LC25000 Borkowski et al. [59] which contains histopathological images of the lungs and colon. The dataset is organised into five classes: lung adenocarcinomas, lung squamous cell carcinomas, lung benign, colon adenocarcinomas, and colon benign. There are 5000 images for each class in the collection, which encompasses 25,000 lung and colon images with pixel sizes of 768 × 768. We train and test the model on a Windows 10 Personal Computer equipped with NVidia Gforce GTX 1060, having 16 GB of RAM, Intel Ci7 64 bit processor. All the simulations are performed on Keras with Tensorflow at the backend.

#### 4.1.2. Training Procedure and Performance of the Model

**Figure 5:**
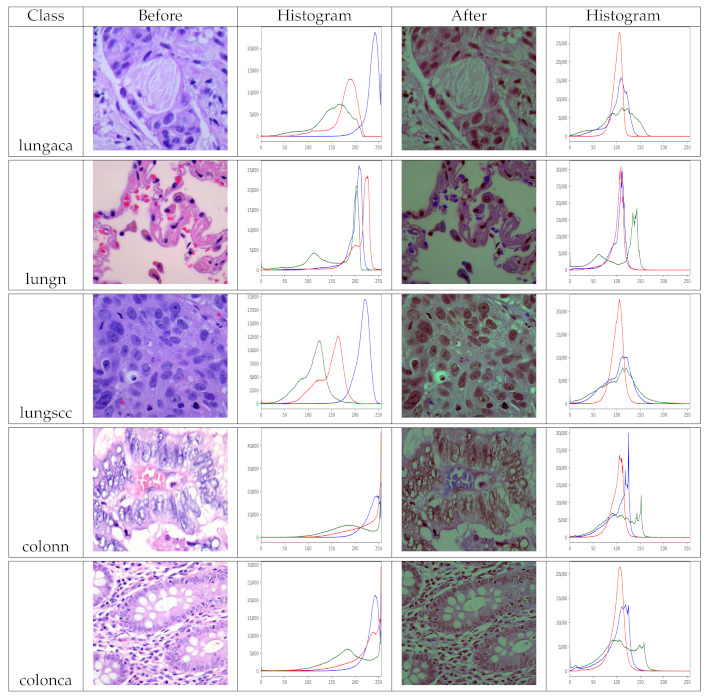
Histograms of original and pre-processed images before and after the pre-processing, lungaca: lung adenocarcinomas, lungscc: lung squamous cell carcinomas, lungn: lung benign, colonca: colon adenocarcinomas, and colonn: colon benign.

Because our model needs two inputs, one with original images and the other with pre-processed images, pre-processing the dataset is a must before training. As a result, we use the pre-processing method described in Section 3.3 to prepare the dataset. The images in the dataset have been pre-processed for each class so that they can benefit as much as possible. Remember that the dataset has two identical versions, one original and one pre-processed. We view the histogram of the images before and after pre-processing to analyze the effect of pre-processing, as shown in Figure 5. The pre-processed version of the images has more balanced color values than the original images, as seen in the depicted histograms, making the information in the image more evident. Finally, multiple inputs aid the model’s feature learning.

We partitioned the dataset into three sections: training, validation and testing, because the training and testing stages of a deep learning model are very critical. From a pool of 25,000 images, we randomly choose 18,320 for training, and the remaining images are randomly assigned to 4580 and 2100 for validation and testing, respectively. The original and pre-processed versions of the dataset have the same distribution. For training procedure, there are certain parameters that must be selected carefully, for instance, learning rate, batch size, number of training epochs, etc. The hyperparameters, optimizer function, proposed loss function along with values for its associated parameters are listed in Table 2.

The process of determining the optimum parameters and their corresponding values for a deep learning model is critical. A variety of strategies are used to assure the models’ robustness [60]. As a result, we use a technique known as *k*-fold cross validation. We utilize the training data, which includes 18,320 samples, to train and test the model *k* times in order to determine the best representation of the model and parameter values. In our technique, we utilize k=5 and keep track of the model’s accuracy for each fold. The model obtains the highest accuracy on the parameters provided in Table 2 during this operation.

**Figure 6:**
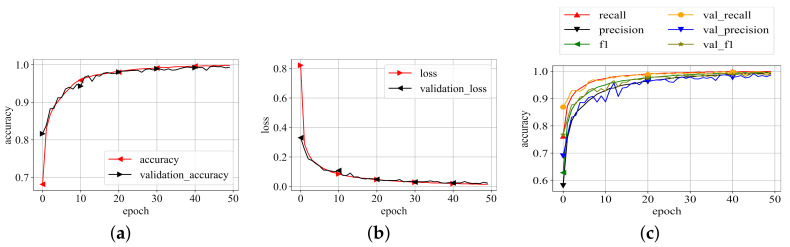
Training results. (**a**) Training accuracy; (**b**) Training loss; (**c**) P, R, f1-scores.

The best representation of the proposed model on the best values of the parameters as listed in Table 2 is then trained on whole training data selected during data distribution and validated on validation data. The model is trained until it converges to its maximum potential. For each epoch of training and validation, the accuracy, loss, precision, recall, and f1-scores were recorded. The related values are shown in Figure 6. Because the results in Figure 6 are averaged for each class, this graph depicts the model’s overall performance on training and validation data. The plots show that the model performs exceptionally well. Aside from loss and accuracy, the precision, recall, and f1-score values are all very promising. The choices of the parameters, particularly the numbers supplied for the threefold loss function, were chosen after an initial trial. The values which produced the best results are listed as the final values.

### 4.2. Performance of the Model on Test Data

For medical image classification, accuracy has often been disregarded as a preferable performance metric. Consider the following scenario: 5% of the training dataset is based on the positive class, and the goal is to classify every case as a negative class. In this case, the model will have a 95% accuracy rate. Despite the fact that the model’s accuracy of 95% on the entire dataset appears to be good, this procedure appears to miss the fact that the model incorrectly categorised all of the positive samples. Because of this, the accuracy is unable to provide appropriate evidence on a model’s functionality within this classification.

Therefore, along with accuracy we also use sensitivity, specificity, f−1 score, and AUC_ROC curves for the performance analysis. As shown in Equation (19), sensitivity is the ratio of True Positive (TP) over (True Positive (TP) + False Negative (FN)). Similarly the specificity in Equation (20) is the ratio of True Negative (TN) over ( True Negative (TN) + False Positive (FP)). We determine the f1-score (24) as $F_{1}=\frac{TP}{TP+\frac{1}{2}\left(FP+FN\right)}$. We get the AUC_ROC curve by plotting the True Positive rate (TPR) compared to the False Positive rate (FPR) at numerous threshold points. In the area of machine learning, the True Positive rate is also recognized as recall, sensitivity, or detection probability. Similarly, the False Positive rate can be calculated as 1−specificity and it is known as the false alarm probability. The performance metrics utilized in this paper are listed below.

(19) $$Sensitivity=\frac{TP}{TP+FN}$$

(20) $$Specificity=\frac{TN}{TN+FP}$$

(21) $$Precision=\frac{TP}{TP+FP}$$

(22) $$Recall=\frac{TP}{TP+FN}$$

(23) $$Accuracy=\frac{TP+TN}{TN+TP+FN+FP}$$

(24) $$F_{1}=\frac{TP}{TP+\frac{1}{2}\left(FP+FN\right)}$$

**Figure 7:**
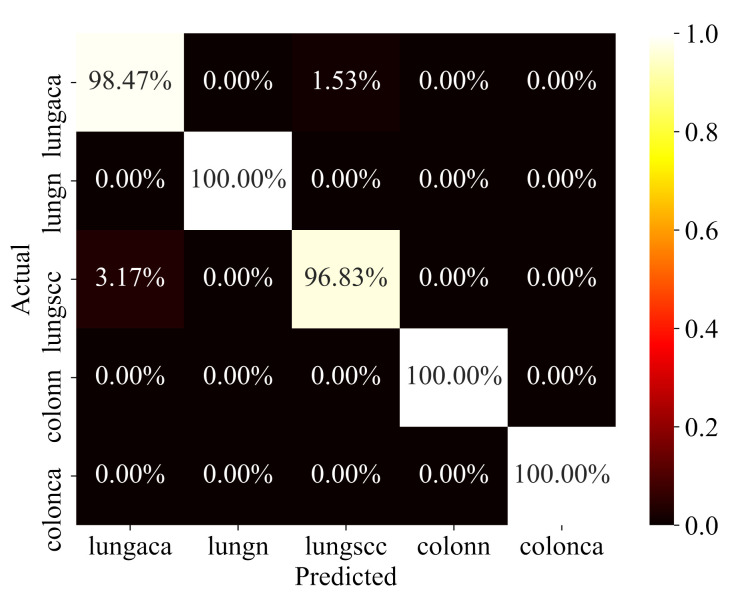
Confusion Matrix.

The proposed model has been rigorously evaluated on test data in order to determine how well it performs on data that has never been seen before. To ensure that the evaluation is fair, the test data was also pre-processed and structured in the same way. The model’s performance on test data is reported in Table 3. The confusion matrix for the same test data is depicted in Figure 7. The model performs well in all categories, but particularly well in identifying colon adenocarcinomas, with 100% prediction accuracy, precision, recall, f1-score, and AUC. Similarly, the model has a 98%, 99%, and 100% accuracy for lung adenocarcinomas, lung squamous cell carcinomas, and lung benign. Sensitivity and specificity are likewise encouraging for all of the classes. In terms of performance, the AUC values for all classes are 100%, which is exceptional.

The key explanation for the improved performance is the richness of the features learned as a result of multiple inputs and varied types of convolutional layer blocks, which allowed the capsule layers to function in a better way. Similarly, better choices of values for threefold margin loss boosted the performance of the proposed model greatly.

To validate the efficacy of the proposed model we systematically compare the acquired results with the results of the state-of-the-art techniques. The proposed model has better performance than state-of-the-art techniques when the lesions of the lungs and the colon are classified simultaneously as shown in Table 4. It can be seen the proposed model has outperformed the state-of-the-art works in terms of precision, recall, f1-score, and accuracy.

### 4.3. Discussion

Deep learning methods for detecting lung and colon cancer use either pre-trained models or convolutional neural networks. However, in addition to the results, the methods can be greatly enhanced. The proposed method in this research uses three common deep learning-based feature extraction mechanisms: convolutional neural networks, separable convolutional neural networks, and capsule networks. As a result, the proposed method greatly reduces the number of trainable parameters while maintaining accuracy. The results show that dual-stream and multi-input, particularly with the color balanced images, have a substantial impact on capsule network performance. Adjusting the red, green, and blue channels of the input images improves the overall performance of deep learning models, especially for histopathology slide images.

Previous research has typically generated lung and colon cancer results separately. They classify lung and colon cancer images using pre-trained models. Lung and colon cancer are treated as two separate classification problems, with each being treated as a binary classification problem. Unlike pre-trained models, we utilize a lightweight model in our method that is based on a small number of parameters. Our method concurrently classifies lung and colon cancer images and treats the problem as a multiclass problem.

## 5. Conclusions

In this research work, we presented a novel multi-input dual-stream capsule network that utilizes powerful feature learning capabilities of conventional and separable convolutional layers to classify histopathological images of lungs and colon cancer into five classes (three malignant and two benign). We trained and tested the proposed model using LC25000 dataset. We pre-processed the dataset with a novel color balancing technique that tries to compensate three color channels before gamma correction and sharpening the prominent features. The proposed model was given two inputs at the same time (one with original images and the second with pre-processed images), which helped the model to learn features in a better way. The produced results show that the model achieved the overall accuracy of 99.58% and f1-score of 99.04%. After comparing the results with the state-of-the-art techniques, we observe that the proposed model performs exceptionally better. By utilizing this model computer-based diagnosing systems can be developed to help pathologists to identify lung and colon cancer cases with less effort, time and cost.

## Figures and Tables

**Table 1:** Summary of the proposed model.

Layer	Channels	Filter Size	Stride	Parameters	Capsules
Convolutional Layer1	64	3×3	1	1792	——
Convolutional Layer2	64	3×3	1	36,928	——
Convolutional Layer3	64	3×3	1	36,928	——
Convolutional layer4	64	3×3	1	36,928	——
Separable Convolutional Layer1	64	3×3	1	137	——
Separable Convolutional Layer2	64	3×3	1	4736	——
Separable Convolutional Layer3	64	3×3	1	4736	——
Separable Convolutional Layer4	64	3×3	1	4736	——
Primary Capsules	——	——	——	5120	48
Class Capsules	——	——	——	——	5

**Table 2:** Hyper parameters and their corresponding values.

Parameter	Value	Parameter	Value
Learning rate	0.0001	$\lambda_{1}$	0.75
Number of epochs	50	$\lambda_{2}$	0.55
Batch size	32	$\lambda_{3}$	0.35
$m_{1}^{+}$	0.90	$m_{1}^{-}$	0.10
$m_{2}^{+}$	0.80	$m_{2}^{-}$	0.20
$m_{3}^{+}$	0.70	$m_{3}^{-}$	0.30
Optimizer	Adam	Input image size	(50,50,3)

**Table 3:** Precision, Recall, f1-score, Accuracy and the number of test images per class, Note: Results are given in percentage.

Class	Accuracy	Precision	Recall	f1-Score	Sensitivity	Specificity	AUC	# Test Images
lungaca	98.90	96.50	98.50	97.50	98.40	99.10	100.00	391
lungn	100.00	100.00	100.00	100.00	100.00	100.00	100.00	417
lungscc	99.00	98.60	96.80	97.70	96.80	96.00	100.00	442
colonn	100.00	100.00	100.00	100.00	100.00	100.00	100.00	424
colonca	100.00	100.00	100.00	100.00	100.00	100.00	100.00	426

**Table 4:** Comparison of the results produced by the proposed model with the state-of-the-art techniques. CNN: Convolutional Neural Networks, CapsNts: Capsule Networks, Note: Results are given in percentage. Sev${}^{*}$: Several pre-trained models were used including VGG16, ResNet50, InceptionV3, InceptionResNetV2, MobileNet, Xception, NASNetMobile, DenseNet169.

Reference	Cancer Type	Image Type	Classifier	Accuracy	Precision	Recall	F-Measure
[48]	Colon	Histopathological	RESNET-50	93.91	95.74	96.77	96.26
[61]	Lung	Histopathological	Sev${}^{*}$	1.00	1.00	1.00	1.00
[61]	Colon	Histopathological	Sev${}^{*}$	1.00	1.00	1.00	1.00
[2]	Lung	Histopathological	CNN	97.89	-	-	-
[2]	Colon	Histopathological	CNN	96.61	-	-	-
[62]	Lung	Histopathological	CNN	97.20	97.33	97.33	97.33
[43]	Lung & Colon	Histopathological	CNN	96.33	96.39	96.37	96.38
Proposed	Lung & Colon	Histopathological	CapsNts	99.58	98.66	99.06	99.04

## Data Availability

The dataset used in this study is publicly available at https://academictorrents.com/details/7a638ed187a6180fd6e464b3666a6ea0499af4af.

## Abbreviations

The following abbreviations are used in this manuscript:

CLB	Convolutional Layers Block
SCLB	Separable Convolutional Layers Block
C	Convolution
SC	Separable Convolution
PC	Pointwise Convolution
DC	Depth-wise Convolution
CNN	Convolutional Neural Networks
